# Piloting and validating the linkage of routine ART and laboratory records in an HIV endemic setting.

**DOI:** 10.23889/ijpds.v7i3.2004

**Published:** 2022-08-25

**Authors:** Dickman Gareta, Dorina Onoya, Kobus Herbst, Jacob Bor

**Affiliations:** 1 Africa Health Research Institute; 2 University of the Witwatersrand, Johannesburg, South Africa; 3 Boston University School of Public Health, Department of Global Health, Boston, MA, United States

## Objectives

Integrating clinical and laboratory data systems offers opportunities to understand disease aetiology and improve patient outcomes. We developed and piloted a data linkage algorithm to link HIV patient treatment records with routine laboratory service records in an HIV endemic setting in rural South Africa.

## Approach

We used data from the South Africa’s National Health Laboratory Service (NHLS) and Three Integrated Electronic Registers (TIER.Net) databases for 17 primary healthcare clinics from uMkhanyakude district, South Africa. The two databases contained data on key demographic variables such as patient first name, surname, date of birth, sex, and health facility, and national ID numbers for a subset of the data. We adapted a probabilistic record linkage algorithm we previously developed for linkage of laboratory results. We trained and validated our algorithm using two approaches: 1) firstly, we constructed a quasi-gold standard based on manual review of potential matches for 1,061 randomly selected patients; 2) secondly, we constructed a quasi-gold standard based on records that contained national ID numbers. We calculated the probabilities that the algorithm could correctly identify a true match (sensitivity) and the probabilities that the match identified by the algorithm was truly a match (positive predictive value) using the two approaches.

## Results

Between 2015 and 2020, 55,077 patients were recorded in the HIV treatment database and 386,577 laboratory tests were recorded in the laboratory service database. The sensitivity and positive predictive value in the manually reviewed data were estimated to be 94.4% and 81.2% respectively. The sensitivity and positive predictive value in the matched individual identifiers were estimated to be 99.6% and 99.7% respectively.

## Conclusion

Records can be linked successfully, but estimated performance of record linkage depends on the validation set used. Manually reviewed data contain noise and may underestimate performance, while national ID numbers may overestimate performance due to non-random patterns of missingness.

